# Production of antiviral “OP7 chimera” defective interfering particles free of infectious virus

**DOI:** 10.1007/s00253-023-12959-6

**Published:** 2024-01-13

**Authors:** Lars Pelz, Tanya Dogra, Pavel Marichal-Gallardo, Marc Dominique Hein, Ghada Hemissi, Sascha Young Kupke, Yvonne Genzel, Udo Reichl

**Affiliations:** 1 Max Planck Institute for Dynamics of Complex Technical Systems, Bioprocess Engineering, Magdeburg, Germany; 2 Otto Von Guericke University Magdeburg, Bioprocess Engineering, Magdeburg, Germany

**Keywords:** Influenza A virus, Defective interfering particles, Antiviral, Cell culture, Perfusion, Alternating tangential flow filtration

## Abstract

**Abstract:**

Defective interfering particles (DIPs) of influenza A virus (IAV) are suggested for use as broad-spectrum antivirals. We discovered a new type of IAV DIP named “OP7” that carries point mutations in its genome segment (Seg) 7 instead of a deletion as in conventional DIPs (cDIPs). Recently, using genetic engineering tools, we generated “OP7 chimera DIPs” that carry point mutations in Seg 7 plus a deletion in Seg 1. Together with cDIPs, OP7 chimera DIPs were produced in shake flasks in the absence of infectious standard virus (STV), rendering UV inactivation unnecessary. However, only part of the virions harvested were OP7 chimera DIPs (78.7%) and total virus titers were relatively low. Here, we describe the establishment of an OP7 chimera DIP production process applicable for large-scale production. To increase total virus titers, we reduced temperature from 37 to 32 °C during virus replication. Production of almost pure OP7 chimera DIP preparations (99.7%) was achieved with a high titer of 3.24 log_10_(HAU/100 µL). This corresponded to an 11-fold increase relative to the initial process. Next, this process was transferred to a stirred tank bioreactor resulting in comparable yields. Moreover, DIP harvests purified and concentrated by steric exclusion chromatography displayed an increased interfering efficacy in vitro. Finally, a perfusion process with perfusion rate control was established, resulting in a 79-fold increase in total virus yields compared to the original batch process in shake flasks. Again, a very high purity of OP7 chimera DIPs was obtained. This process could thus be an excellent starting point for good manufacturing practice production of DIPs for use as antivirals.

**Key points:**

• *Scalable cell culture-based process for highly effective antiviral OP7 chimera DIPs*

• *Production of almost pure OP7 chimera DIPs in the absence of infectious virus*

• *Perfusion mode production and purification train results in very high titers*

**Supplementary Information:**

The online version contains supplementary material available at 10.1007/s00253-023-12959-6.

## Introduction

Influenza A virus (IAV) is a respiratory pathogen, which contains a negative-sense, single-stranded RNA genome with eight viral RNA (vRNA) segments (Krammer et al. 2018). Each year, IAV infections lead to a high disease burden with up to 290,000–650,000 deaths globally (WHO 2023). Current preventive measures include annual flu vaccination and the use of antivirals. However, the update in the composition of seasonal vaccines for the northern and southern hemisphere is a time-consuming process that involves the risk of poor vaccine effectiveness due to a mismatch of vaccine strains (reviewed by Chen et al. (Chen et al. 2021)). Moreover, the use of presently available antivirals like neuraminidase or M2 ion channel inhibitors has resulted in the emergence of resistant IAV strains (Chen et al. 2021). Consequently, further options for flu disease prevention and treatment would be highly appreciated.

Defective interfering particles (DIPs) of IAV are naturally occurring viral mutants that inhibit infectious standard virus (STV) propagation of IAV (Dimmock et al. 2008; Hein et al. 2021a; Huo et al. 2020; Pelz et al. 2021; Zhao et al. 2018). In addition, DIPs with antiviral activity exist for many other virus families (Chaturvedi et al. 2021; Levi et al. 2021; Rezelj et al. 2021; Smither et al. 2020; Welch et al. 2020). Therefore, DIPs were suggested as promising antivirals (Bdeir et al. 2019; Frensing 2015; Genoyer and Lopez 2019; Karki et al. 2022; Vasilijevic et al. 2017). Conventional IAV DIPs (cDIPs) contain a large internal deletion in one of the eight vRNAs (Dimmock and Easton 2015). The short defective interfering (DI) vRNAs are believed to replicate faster than the parental full-length (FL) vRNA in a co-infection with STV, thereby drawing away cellular and viral resources from STV (i.e., “replication inhibition”) (Laske et al. 2016; Marriott and Dimmock 2010; Nayak et al. 1985; Rüdiger et al. 2021). As a result, IAV DIPs can suppress a variety of IAV strains including epidemic and pandemic human, and even highly pathogenic avian IAV as shown in in vitro and in animal experiments (Dimmock et al. 2008, Dimmock et al. 2012b; Huo et al. 2020; Kupke et al. 2019; Zhao et al. 2018). Simultaneously, they strongly stimulate the interferon (IFN)-induced antiviral activity against IAV infections (Frensing et al. 2014; Huo et al. 2020; Penn et al. 2022). Furthermore, this unspecific innate immune response stimulation can also suppress replication of unrelated viruses including severe acute respiratory syndrome coronavirus (SARS-CoV-2) (Easton et al. 2011; Pelz et al. 2023; Rand et al. 2021; Scott et al. 2011). Previously, we discovered a new type of IAV DIP named “OP7”. OP7 harbors multiple nucleotide substitutions in segment (Seg) 7 vRNA instead of the large internal deletion of cDIPs. The 37 point mutations involve promoter regions, genome packaging signals, and encoded proteins (Kupke et al. 2019). Relative to cDIPs, OP7 exhibited an even higher interfering efficacy in vitro and in vivo, highlighting its potential for use as an antiviral (Hein et al. 2021a, 2021c; Rand et al. 2021).

**Fig. 1:**
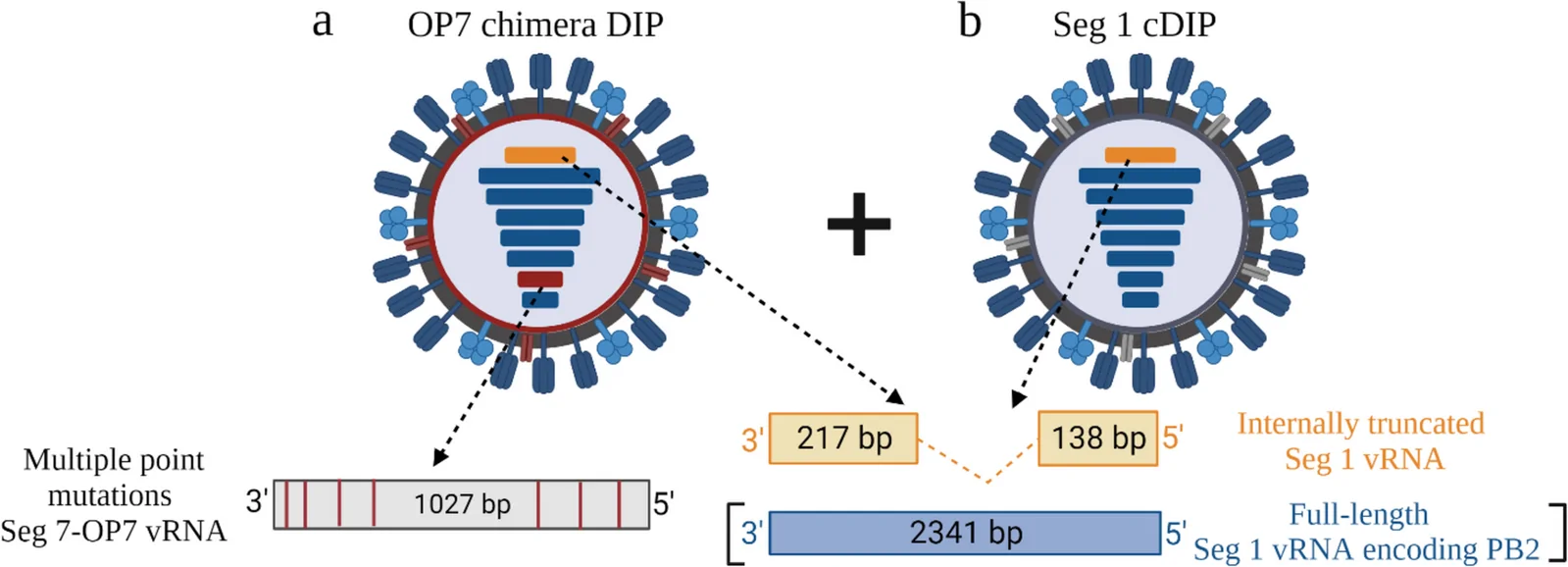
Scheme of OP7 chimera DIPs and Seg 1 cDIPs. Reverse genetics was used to generate a mixture of **a)** OP7 chimera DIPs and **b)** Seg 1 cDIPs. Both together replicate without addition of infectious standard virus as the missing PB2 is provided by MDCK-PB2(sus) cells. Figure adapted from (Dogra et al. 2023). Created with BioRender.com

Recently, we established a cell culture-based production system for “OP7 chimera DIPs” that harbor both, nucleotide substitutions in Seg 7 vRNA plus a large internal deletion in Seg 1. In the presence of cDIPs, the addition of STV is not required for their propagation, and DIP harvests do not contain any infectious material. This renders UV inactivation unnecessary and alleviates safety and regulatory concerns with respect to medical application (Dogra et al. 2023). For this, we modified a reverse genetics workflow (Bdeir et al. 2019; Hein et al. 2021a) and reconstituted a population of two types of DIPs: OP7 chimera DIPs (Fig. 1a) and Seg 1 cDIPs (Fig. 1b). OP7 chimera DIPs harbor Seg 7 of OP7 (Seg 7-OP7) vRNA, a truncated Seg 1 vRNA, and the remaining six FL vRNAs. Seg 1 cDIPs contain a deletion in Seg 1 vRNA and seven FL vRNAs. To complement for the defect in virus replication, suspension Madin-Darby canine kidney (MDCK) cells were genetically engineered that express the viral polymerase basic 2 (PB2) protein (encoded on Seg 1) (Bdeir et al. 2019; Hein et al. 2021a) and are used for cell culture-based production. First results with OP7 chimera DIP material harvested from shake flasks suggest a high tolerability and high antiviral efficacy after intranasal administration in mice. These initial experiments, however, only resulted in relatively low total virus titers with OP7 chimera DIP fractions of 78.7% (Dogra et al. 2023).

In the present work, we developed a scalable laboratory-scale process in a stirred tank bioreactor (STR) for high-yield production of almost pure OP7 chimera DIPs preparations. In perfusion mode, we even achieved a 79-fold increase in total virus yields compared to the original batch process in shake flasks. Together with a steric exclusion-based chromatographic purification train, this process may be adopted towards good manufacturing practice (GMP) production for safety and toxicology studies and clinical trials.

## Materials and methods

### Cells and viruses

MDCK cells growing in suspension culture (Lohr et al. 2010) and stably expressing PB2 (encoded by Seg 1) (Bdeir et al. 2019; Hein et al. 2021b), referred to as MDCK-PB2(sus) cells, were used. These cells were cultivated in Xeno™ medium (Shanghai BioEngine Sci-Tech) supplemented with 8 mM glutamine and 0.5 μg/mL puromycin as selection antibiotic and maintained in shake flasks (125 mL baffled Erlenmeyer flask with vent cap, Corning, #1356244) in 50 mL working volume (V_W_). Cultivations of cell cultures were performed in an orbital shaker (Multitron Pro, Infors HT; 50 mm shaking orbit) at 185 rpm, 37 °C, and 5% CO_2_ environment. MDCK(adh) cells grew in Glasgow Minimum Essential Medium (GMEM) supplemented with 10% fetal bovine serum (FBS, Merck, #F7524) and 1% peptone (Thermo Fisher Scientific, #211709). For adherent MDCK cells (ECACC, #84121903) expressing PB2 (MDCK-PB2(adh), generated by retroviral transduction, as described in (Bdeir et al. 2019)), the medium was supplemented with 1.5 μg/mL puromycin. Human alveolar epithelial Calu-3 cells were provided by Dunja Bruder (Helmholtz Centre for Infection Research, Braunschweig, Germany) and cultivated in Minimum Essential Medium (MEM) with 10% FBS, 1% penicillin/streptomycin, and 1% sodium pyruvate at 37 °C and 5% CO_2_. Viable cell concentration (VCC), viability, and cell diameter were quantified by a cell counter (Vi-Cell™ XR, Beckman coulter). Metabolite concentrations (glucose, lactate, glutamine, ammonium) were quantified with a Cedex Bio® Analyzer (Roche).

IAV strain A/PR/8/34 H1N1 (STV PR8) was provided by the Robert Koch Institute (Berlin, Germany, #3138) (seed virus: infectious virus titer 1.1 × 10^9^ 50% tissue culture infectious dose ((TCID_50_)/mL). The OP7 chimera DIP seed virus (4.5 × 10^6^ plaque-forming units ((PFU)/mL) was previously produced in MDCK-PB2(sus) cells in batch mode after a complete medium exchange (CME) in shake flasks at a multiplicity of infection (MOI) of 10^–4^ at 37 °C (Dogra et al. 2023). MOIs reported in the following are based on the TCID_50_ titer (Genzel and Reichl 2007) (interference assay) or the plaque titer (OP7 chimera DIP production).

### Production of OP7 chimera DIPs in shake flasks

For infection experiments in shake flasks, 250-mL shake flasks (baffled Erlenmeyer flask with vent cap, Corning, #1356246) with 100 mL V_W_ were used. To produce OP7 chimera DIPs in batch mode, cells were infected at 2.0 × 10^6^ cells/mL either by direct inoculation after a CME, or by 1:2 dilution with fresh medium (MD) of a culture grown to 4.0 × 10^6^ cells/mL. Production with CME was performed as described recently (Hein et al. 2021a). In brief, MDCK-PB2(sus) cells in exponential growth phase were centrifuged (300 × g, 5 min, room temperature (RT)). The cell pellet was resuspended in fresh medium (without puromycin) containing trypsin (final activity 20 U/mL, Thermo Fisher Scientific, #27250–018). Subsequently, cells were seeded into shake flasks and infected at a MOI of 10^–4^ at about 2.0 × 10^6^ cells/mL at 37 °C. For production with MD, cells were centrifuged (300 × g, 5 min, RT) and resuspended at 0.6 × 10^6^ cells/mL in fresh medium (without puromycin). Next, cells were cultivated up to about 4.0 × 10^6^ cells/mL and then diluted (1:2) with fresh medium containing trypsin (final activity of 20 U/mL) for subsequent infection at 37 °C or at 32 °C using indicated MOIs. For sampling, aliquots of cell suspensions were centrifuged (3000 × g, 4 °C, 10 min) and supernatants were stored at -80 °C until further analysis. From these supernatants, vRNAs of progeny virions were purified using the NucleoSpin RNA virus kit (Macherey–Nagel, #740956) according to the manufacturers’ instructions, and stored at -80°C until real-time reverse transcription-quantitative PCR (real-time RT-qPCR).

### Batch mode production of OP7 chimera DIPs in a STR

Cells grown in shake flasks were centrifuged (300 × g, 5 min, RT), resuspended in fresh puromycin-free medium and used to inoculate a 1 L STR (DASGIP® Parallel Bioreactor System, Eppendorf AG, #76DG04CCBB) at 0.5 × 10^6^ cells/mL (400 mL V_W_). The STR was equipped with an inclined blade impeller (three blades, 30° angle, 50 mm diameter, 150 rpm) and a L-macrosparger. A mixture of air and oxygen was provided to control the dissolved oxygen above 40% air saturation. pH control (pH 7.6, reduced to 7.4 as soon set point pH 7.6 could no longer be maintained) was achieved by CO_2_ sparging and addition of 7.5% NaHCO_3_. During the cell growth phase, temperature was set to 37 °C and cells were grown to about 4.0 × 10^6^ cells/mL. Prior to infection, temperature was reduced to 32 °C, MD (1:2 dilution with fresh medium) was performed (final V_W_ about 700 mL) and cells were infected at a MOI of 10^–4^ and pH of 7.4.

### Production of OP7 chimera DIPs in a STR in perfusion mode

An alternating tangential flow filtration (ATF2) system with C24U-v2 controller (Repligen), equipped with a hollow fiber membrane (polyethersulfone (PES), 0.2 μm pore size, Spectrum Labs) was coupled to the 1 L STR described above (final V_W_ about 700 mL) for perfusion cultivation. Cells were inoculated at 1.2 × 10^6^ cells/mL and cultivated for 1 day in batch mode. Subsequently, perfusion was started and the recirculation rate was set to 0.9 L/min. For perfusion rate control, a capacitance probe connected to an ArcView Controller 265 (Hamilton) was utilized (Göbel et al. 2023; Gränicher et al. 2021; Hein et al. 2021b, 2023; Nikolay et al. 2018; Wu et al. 2021). Using linear regression, the permittivity signal was converted to the VCC and used to control the permeate flow rate of a connected peristaltic pump (120 U, Watson-Marlow). The cell factor in the ArcView controller was re-adjusted after every sample taking to keep a cell-specific perfusion rate (CSPR) of 200 pL/cell/day as described previously (Hein et al. 2021b). The feed flow rate was controlled based on the weight of the bioreactor. Prior to infection, one reactor volume (RV) was exchanged with fresh medium and temperature was lowered to 32 °C. After infection at a MOI of 10^–4^, the permeate flow rate was set to 0 RV/day for 1 h, kept constant at 2.4 RV/day until 30 h post infection (hpi) and finally increased to 2.6 RV/day. In order to prevent oxygen limitation during cell growth phase (Hein et al. 2021b), 0.5 L/h of air was provided 77.2 h after inoculation using an additional microsparger.

### Membrane-based steric exclusion chromatography

Harvested OP7 chimera DIP material was clarified (3000 × g, 10 min, 4 °C) and spiked with sucrose (5%, 84097, Merck). Next, consecutive filtration steps with regenerated cellulose membranes (1.0 μm, #10410014; 0.45 μm, #10410214; 0.2 μm, #10410314, Cytiva) were performed for clarification using a bottle top system coupled to a vacuum pump. To remove host cell DNA, the clarified OP7 chimera DIP material was supplemented with MgCl_2_ (2 mM final concentration, #M8266, Merck) and treated with an unspecific nuclease (40 U/mL final activity, Denarase®, #2DN100KU99, Sartorius Stedim Biotech) for 4 h under mixing. Purification was done by membrane-based steric exclusion chromatography (SXC) (Marichal-Gallardo et al. 2017, Marichal-Gallardo et al. 2021) as described recently (Hein et al. 2021a, 2021c). An ÄKTA Pure 25 system (Cytiva) was used for chromatography at RT. UV monitoring was performed at 280 nm and virus particles were monitored using a NICOMPTM 380 (Particle Sizing Systems) at 632.8 nm. The filter unit (in the following referred to as “column”) was packed with regenerated cellulose membranes (1.0 μm pore size, 20 layers, 100 cm^2^ total surface) and installed in a 25 mm stainless steel filter housing. The flow rate was 10 mL/min. For equilibration, the column was washed with water and then with binding buffer (8% PEG-6000 in PBS, #81260, Merck). Next, the sample was injected (in-line mixing with 16% PEG-6000 in PBS to achieve 8% PEG-6000). Subsequently, the column was washed with binding buffer until baseline UV absorbance was reached. Elution was conducted with 20 column volumes of elution buffer (PBS). The eluate was dialyzed overnight at 4 °C against PBS (sample to buffer ratio of 1:1000) using cellulose ester dialysis tubing (300 kDa cut-off, #GZ-02890–77). Subsequently, the material was spiked with sucrose (5%). Finally, the material was sterile filtered (0.2 μm, cellulose acetate syringe filter, #16534-K, Sartorius Stedium Biotech).

### Virus quantification

Real-time RT-qPCR was used to quantify purified vRNAs of progeny virions as described previously (Kupke et al. 2019). Primers used for quantification of the vRNA of Seg 7-OP7 are listed in (Hein et al. 2021c) and, for Seg 7 of the wild-type (WT) virus (Seg 7-WT), in (Dogra et al. 2023). The plaque assay was carried out to quantify infectious virus titers with MDCK(adh) cells (interference assay) and MDCK-PB2(adh) cells (seed virus titer of OP7 chimera DIP preparation) as described previously (Hein et al. 2021a, 2021c; Kupke et al. 2020) with a measurement error of ± 0.2 log_10_. A hemagglutination assay (HA assay) was used to determine total virus titers (log_10_(HAU/100 µL)) with a measurement error of ± 0.15 log_10_(HAU/100 µL) (Kalbfuss et al. 2008).

The accumulated HA titer (log_10_(HAU/100 µL)) was estimated from the HA titer of the harvest in the bioreactor vessel plus the virus particles collected after the hollow fiber membrane (detected in the permeate line) and quantified according to Eq. 1. HA_B_ denotes the HA titer of the sample taken at the optimal harvest time point in the bioreactor vessel, V_W_ (mL) of the bioreactor vessel, HA_P_ the average HA titer of material collected in the permeate line between the sample time point t_n_ and the previous sample time point t_n-1_ with the harvested volume (V_p_).

1 $${HA}_{acc}={log}_{10}\frac{{10}^{{HA}_{B}} \times {V}_{W} + \sum {(10}^{{HA}_{P}} \times {V}_{P})}{{V}_{W}}$$

The concentration of DIPs (c_DIP_, virions/mL) was calculated using Eq. 2, where c_RBC_ denotes the concentration of red blood chicken cells used in the HA assay (2.0 × 10^7^ cells/mL).

2 $${c}_{DIP}={10}^{{log}_{10}(\frac{HAU}{100 \mu L})}\times {c}_{RBC}$$

The total number of produced virus particles vir_tot_ (virions) was determined according to Eq. 3. c_B_ denotes the c_DIP_ in the bioreactor vessel at the optimal harvest time point, and c_p_ the average c_DIP_ in the permeate line between t_n_ and t_n-1_.

3 $${vir}_{tot}={c}_{B}\times {V}_{W}+\sum {c}_{P}\times {V}_{P}$$

The cell-specific virus yield (CSVY, virions/cell) was calculated using Eq. 4, where VCC_max_ (cells/mL) denotes the maximum VCC after time of infection (TOI).

4 $$CSVY=\frac{{vir}_{tot}}{{VCC}_{max} \times {V}_{W}}$$

The space–time yield (STY, virions/L/day) was determined using Eq. 5. t_tot_ (day) denotes the total time from inoculation until the optimal harvest time point.

5 $$STY=\frac{{vir}_{tot}}{{V}_{W} \times {t}_{tot}}$$

The volumetric virus productivity (VVP, virions/L/day) was estimated according to Eq. 6, where V_tot_ denotes the total volume of the spent medium during cell growth and virus production phase.

6 $$VVP=\frac{{vir}_{tot}}{{V}_{tot} \times {t}_{tot}}$$

The percentage of virus particles that passed the pores of the hollow fibers (P_Perm_, %) was determined according to Eq. 7. *n* denotes the total number of sample time points, HA_P_ the HA titer in the permeate line at t_n_, and HA_B_ the HA titer in the bioreactor vessel at t_n_.

7 $${P}_{Perm}= \frac{1}{n} \sum \left(\frac{{10}^{{HA}_{P}}}{{10}^{{HA}_{B}}}\right)\times 100\%$$

### Interference assay

To determine the in vitro interfering efficacy of the produced OP7 chimera DIP material, an interference assay was used. Specifically, we evaluated the inhibition of STV propagation after co-infection with OP7 chimera DIPs. Co-infections were performed in MDCK(adh) cells (Hein et al. 2021a, 2021c) or in Calu-3 cells. Calu-3 cells were seeded at a concentration of 3.0 × 10^6^ cells/well in a 12-well plate and incubated for 24 h prior to infection. For infection, cells were washed with PBS and infected with STV PR8 at a MOI of 0.05 or co-infected with 125 µL of the produced OP7 chimera DIP material in a total volume of 250 µL of media. After 1 h, we filled up to 2 mL with medium. Supernatants were harvested at indicated time points, centrifuged at 3000 × g for 10 min at 4 °C and cell-free supernatants stored at -80°C until virus quantification. To extract intracellular RNAs, 350 µL of RA1 buffer (Macherey Nagel, #740961), 1% β-mercaptoethanol was added to cells remaining in wells for lysis. RNA purification from these lysates was carried out according to the manufacturer’s instructions and samples were stored at -80°C until real-time RT-qPCR to monitor IFN-β gene expression as described previously (Kupke et al. 2019; Rand et al. 2021). Fold changes were calculated using the ΔΔc_T_ method.

### Statistical analysis and data visualization

GraphPad Prism 9 (GraphPad Software) was used for statistical analysis and data visualization. Either one-way analysis of variance (ANOVA) followed by Tukey´s multiple comparison test, two-way ANOVA followed by Dunnett´s multiple comparison test, or unpaired t test were used to determine significance.

## Results

### Medium dilution impairs yields, whereas infection at 32 °C increases OP7 chimera DIP titers and fractions in shake flasks

Previously, a CME prior to infection has been performed for cell culture-based production of OP7 chimera DIPs (Dogra et al. 2023). However, this is difficult to implement at larger scales without cell retention devices. Therefore, following a cell growth phase until about 4.0 × 10^6^ cells/mL, we added fresh medium (MD, 1:2 dilution) to supply substrates and reduce the level of inhibitors accumulated as by-products. To investigate whether a reduction in temperature has a positive effect on virus replication and yields (Hein et al. 2021b; Wu et al. 2021), two cultivations were performed at 37 °C and 32 °C with MD; in addition, one cultivation at 37 °C with CME was performed as a control.

**Fig. 2:**
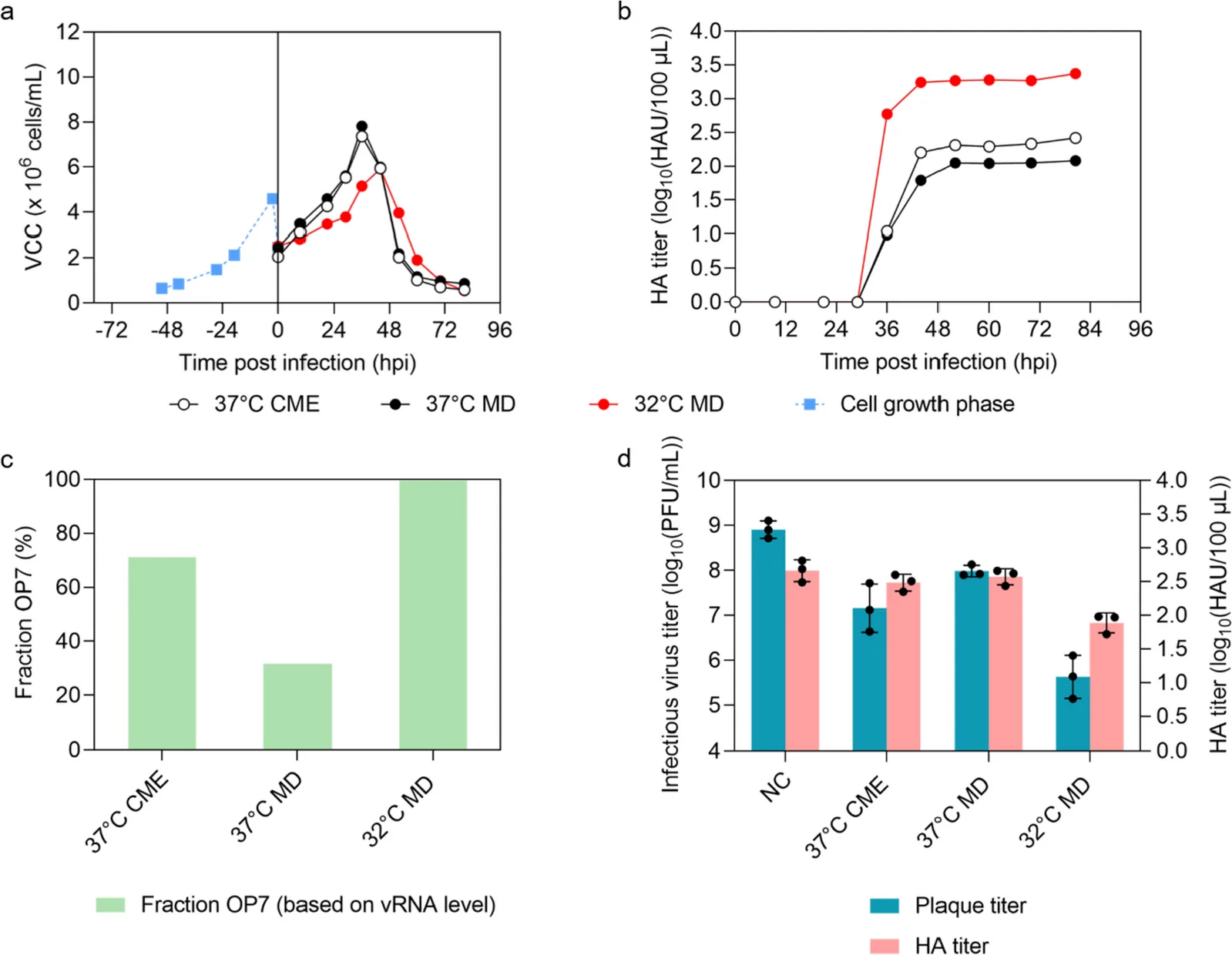
Batch mode production of OP7 chimera DIPs in shake flasks with MD and temperature decrease to 32 °C. MDCK-PB2(sus) cells, cultivated in 250 mL shake flasks (100 mL V_W_) at 37 °C, were grown to about 4.0 × 10^6^ cells/mL. Subsequently, the suspension culture was diluted (MD, 1:2) with fresh medium (100 mL V_W_), cells were infected at a MOI of 10^–4^ and temperature was reduced to 32 °C. For comparison, two cultivations were performed at 37 °C, one with MD at an optimal MOI of 10^–3^, one with CME at an optimal MOI of 10^–4^. **a)** VCC. **b)** HA titer. **c)** Fraction of OP7 chimera DIPs, calculated using the extracellular Seg 7-OP7 and Seg 7-WT vRNA concentrations, quantified by real-time RT-qPCR. **a-c** depict the results of one experiment. **d)** Interference assay. WT MDCK(adh) cells were infected with STV PR8 at a MOI of 10 (negative control, NC) or co-infected with 125 μL of indicated OP7 chimera DIP material. Infectious virus particle release is indicated by the plaque titer and total virus particle release by HA titer (16 hpi). Interference assay was performed in three independent experiments. Error bars indicate standard deviation (SD). The optimal harvest time point (37 °C CME: 44 hpi, 37 °C MD: 52 hpi, 32 °C MD: 44 hpi) was analyzed for **c** and **d**

The infection with MD at 37 °C resulted in a similar VCC dynamics relative to the production with CME (Fig. 2a). However, a slightly lower maximum HA titer of 2.05 log_10_(HAU/100 µL) compared to 2.20 log_10_(HAU/100 µL) was found (Fig. 2b). The lower total virus titer is likely associated with an increased ammonium (inhibitor) concentration and a depletion of glutamine during the infection phase (Fig. S1). A low OP7 chimera DIP fraction of 31.6% (MD) relative to 71.2% (CME) was reached (Fig. 2c, based on the extracellular vRNA concentration of Seg 7-OP7 and Seg 7-WT quantified by real-time RT-qPCR). Lowering the temperature to 32 °C before infection counterbalanced this negative effect of MD and resulted in higher HA titers (Fig. 2b). Here, a maximum of 3.24 log_10_(HAU/100 µL) was observed at 44 hpi corresponding to an 11-fold increase relative to the production at 37 °C with CME. In addition, virus production at 32 °C resulted in reduced concentrations of ammonium (< 3.6 mM, Fig. S1), which should also favor IAV propagation. Finally, the OP7 chimera DIP fraction was greatly increased to 99.7% (Fig. 2c), an almost pure OP7 chimera DIP preparation. To demonstrate reproducibility of this optimized production, a second production run was carried out subsequently (Fig. S2) that confirmed these findings.

Next, we tested the interfering efficacy of the produced material in vitro, in which we assessed the inhibition of STV PR8 replication during co-infection with different produced OP7 chimera DIP materials. For this, we used samples at the respective optimal harvest time points (37 °C CME: 44 hpi, 37 °C MD: 52 hpi, 32 °C MD: 44 hpi) (Fig. 2d). Here, the HA titer almost plateaued and biological activity of the virus particles sampled is assumed highest before onset of unspecific degradation over time (Genzel et al. 2010). For material produced at 32 °C and MD, we observed a strong reduction of the infectious STV PR8 titer (more than three orders of magnitude), which was significantly different to the small reduction observed for material produced at 37 °C and MD (*p* < 0.001, one-way ANOVA followed by Tukey’s multiple comparison test). In addition, the reduction of the infectious virus titer was significantly higher than for material produced at 37 °C and CME (*p* < 0.01). Overall, this confirms a high interfering efficacy of OP7 chimera DIP preparations produced at 32 °C and MD. Regarding the total virus particle release, as expressed by the HA titer, this trend was less pronounced.

Previous studies suggested clearly that OP7 chimera DIP production and interfering efficacy strongly depend on the MOI (Dogra et al. 2023). Therefore, only productions performed at the optimal MOIs were shown in Fig. 2. Interestingly, however, MOI dependency on total virus titers, OP7 chimera DIP fraction and interfering efficacy was negligible for MD and 32 °C (Fig. S3).

In summary, the optimized production at 32 °C with MD (1:2) resulted in an increase of total virus yields by 11-fold compared to the previous processes operated at 37 °C and CME. In addition, a production of almost pure OP7 chimera DIP preparation was achieved.

### Batch mode production of OP7 chimera DIPs in a bioreactor and purification by SXC

In order to show that production of OP7 chimera DIPs at larger scale is possible, the process was transferred to a STR with 700 mL V_W_. Three independent productions were performed (STR 1, STR 2, and STR 3) and compared to two productions in shake flasks (SF 1 (Fig. 2), and SF 2 (Fig. S2)).

**Fig. 3:**
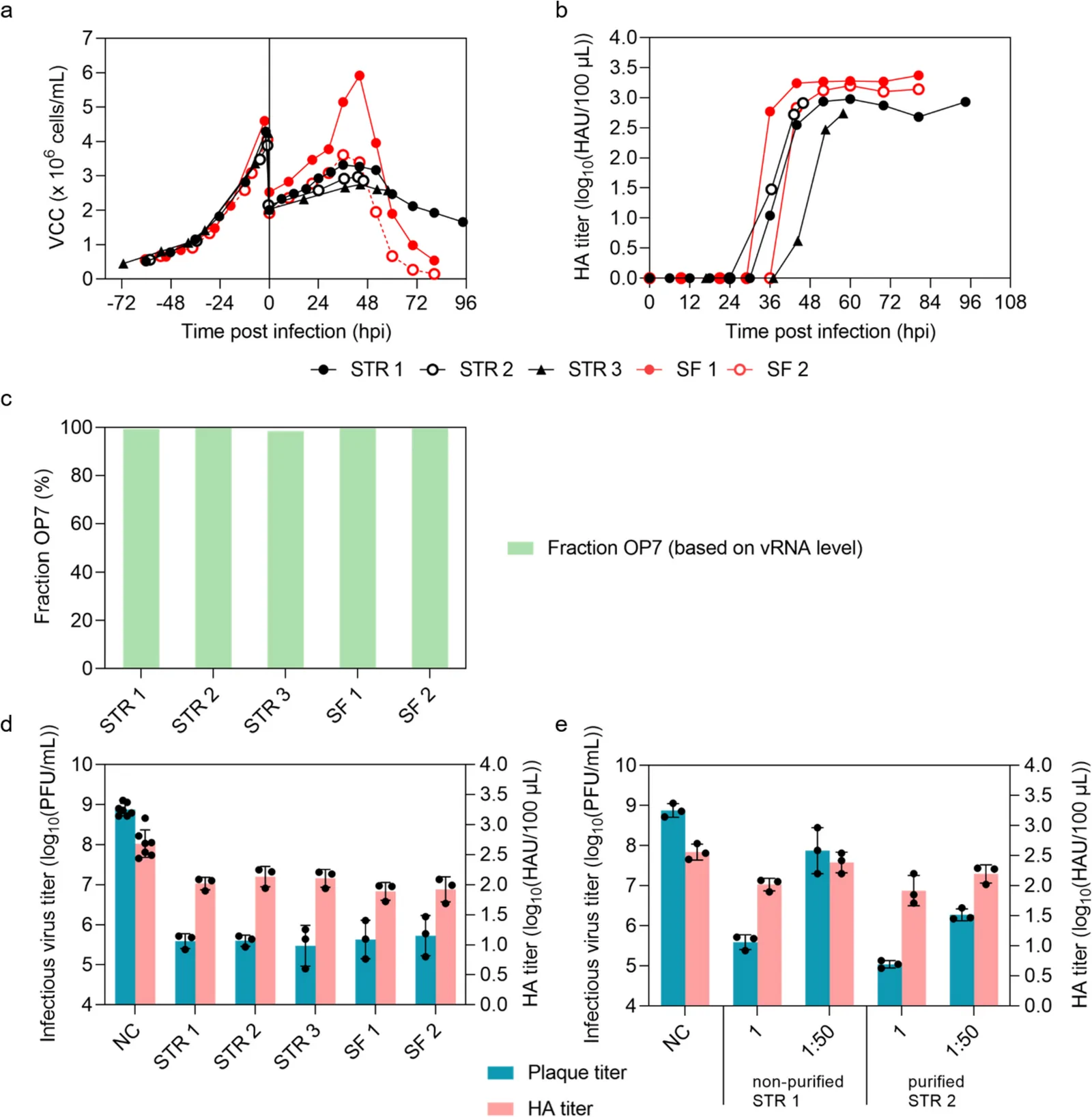
OP7 chimera DIP production in batch mode in a 1 L STR versus shake flask production and interfering efficacy of produced material after purification by SXC. MDCK-PB2(sus) cells were grown in a 1 L STR (400 mL V_W_, 37 °C). After growth to about 4.0 × 10^6^ cells/mL, cells were diluted (1:2) with fresh medium (approx. 700 mL final V_W_), temperature was set to 32 °C, pH was set to 7.4, and cells were infected at a MOI of 10^–4^. In total, three independent productions were carried out in STR (STR 1**–**3) and compared to two independent productions in shake flasks (SF 1**–**2, 100 mL V_W_) for comparison (also shown in Fig. 2 and Fig. S2). **a)** VCC. **b)** HA titer. **c)** Fraction of OP7 chimera DIPs (see Fig. 1). **d)** Interference assay with MDCK(adh) cells was performed in seven independent experiments for NC, and three experiments for STRs and SFs. **e)** Interference assay with MDCK(adh) cells of purified (SXC) vs non-purified material. Dilutions of the tested materials are indicated. Interference assay was performed in three independent experiments. Error bars indicate the SD. The optimal harvest time point (STR 1: 52 hpi, STR 2: 46 hpi, STR 3: 58 hpi, SF 1: 44 hpi, SF 2: 44 hpi) was analyzed for **c**, **d** and **e**

MDCK-PB2(sus) cells were seeded into the STR at approx. 0.5 × 10^6^ cells/mL and cultivated (400 mL, 37 °C) until a VCC of about. 4.0 × 10^6^ cells/mL (Fig. 3a) was obtained. As in SF, cultivations were performed at 32 °C and MD (1:2) (final V_W_ about 700 mL). Cells were infected at a MOI of 10^–4^. After infection, cells continued to grow (Fig. 3a), with STR 1–3 and SF 2 showing very similar growth curves before onset of virus-induced cell lysis (max. VCC 2.8–3.6 × 10^6^ cells/mL). SF 1 revealed a peak VCC of 5.9 × 10^6^ cells/mL likely due to a more rapid growth and higher VCC at TOI. HA titers were similar (*p* > 0.05, unpaired t test) (Fig. 3b) for all STR runs compared to SF productions. (Note that STR 2 and 3 were terminated at 46 hpi and 58 hpi, respectively, for virus harvest.) In addition, all cultivations showed very high OP7 chimera DIP fractions (98.5–99.7%) at the optimal harvest time point (Fig. 3c). Finally, results from the in vitro interference assay (Fig. 3d) showed no significant difference in the reduction of infectious virus particle release (*p* > 0.05, one-way ANOVA followed by Tukey’s multiple comparison test) and total virus particle release (HA titer) (*p* > 0.05).

For virus purification, material harvested from STR 2 was subjected to SXC. The purified material was tested for antiviral efficacy in comparison to the non-purified material using the in vitro interfering assay (Fig. 3e). There was no significant reduction in infectious virus particle release for purified material (STR 2, 1.1 × 10^5^ PFU/mL) compared to non-purified material (STR 1, 4.1 × 10^5^ PFU/mL) (*p* > 0.05) (Fig. 3d and e). Yet, a higher interfering efficacy of the purified material was found for diluted samples (1:50) that showed a significantly higher decrease in the release of infectious virus particles (STR 2, 2.0 × 10^6^ PFU/mL) compared to the diluted non-purified material (STR 1, 1.3 × 10^8^ PFU/mL) (*p* < 0.001) (Fig. 3e).

**Fig. 4:**
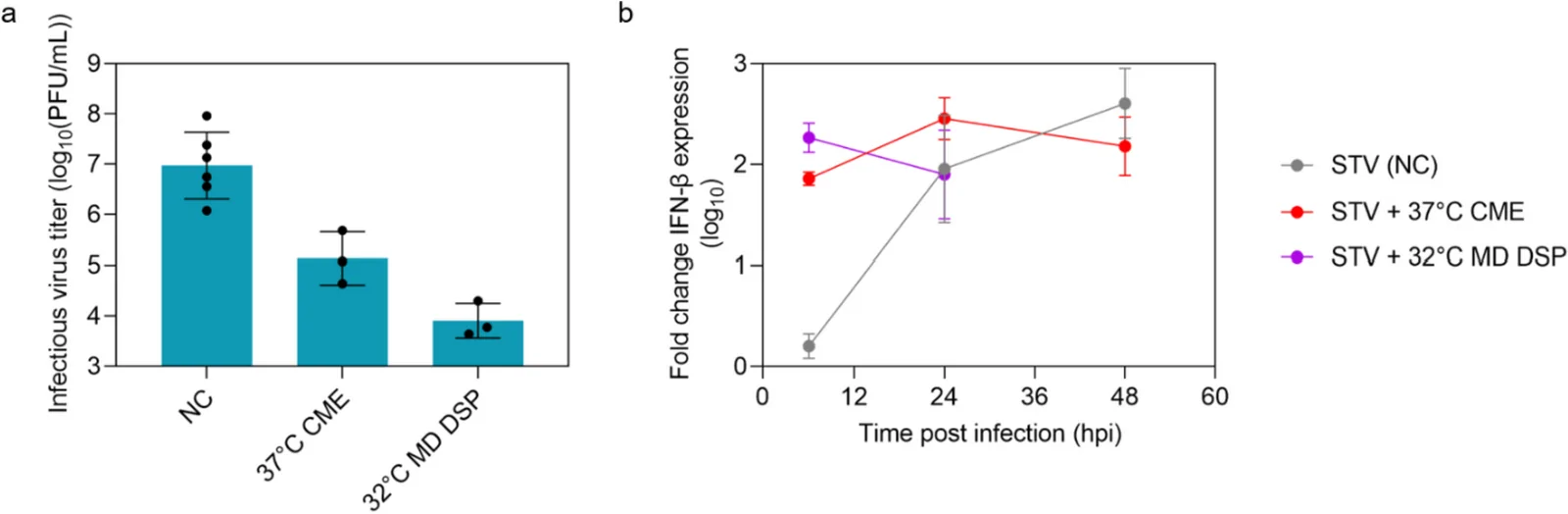
Interfering efficacy in human alveolar epithelial Calu-3 cells of OP7 chimera DIP material purified by SXC. Calu-3 cells were infected with STV PR8 alone at a MOI of 0.05 (NC) or co-infected with 125 μL of indicated OP7 chimera DIP material. **a)** Infectious virus particle release, shown by the plaque titer (24 hpi). **b)** Fold change in IFN-β gene expression, quantified by real-time RT-qPCR. Results of six independent experiments for NC, or three experiments for the co-infection are shown. Material produced in shake flasks at 37 °C with CME (37 °C CME, Fig. 2) was compared to material produced in a STR at 32 °C with MD and further SXC purification (32 °C MD Downstream Processing (DSP), Fig. 3e). Error bars indicate the SD

Next, we investigated the antiviral activity of the purified OP7 chimera DIP material in vitro in human alveolar epithelial (Calu-3) cells (Fig. 4). In contrast to MDCK(adh) cells used for this assay before (Figs. 2d, 3d and e), Calu-3 cells have a functional innate immune response against human IAV (Hsu et al. 2011; Seitz et al. 2010) including an IFN response that induces a cellular antiviral state. Accordingly, MDCK cells were used to only monitor replication inhibition caused by DIP co-infections, whereas the use of Calu-3 cells allowed additional contribution of innate immunity. With the Calu-3 cell assay, we observed a strong suppression of infectious virus particle release (by roughly two orders of magnitude) upon co-infection with non-purified OP7 chimera DIP preparations produced in SF at 37 °C and CME (original process) (Fig. 4a). After process optimization (32 °C MD DSP), including STR production at 32 °C and SXC purification, the preparations appeared to interfere slightly stronger (three instead of two orders of magnitude, Fig. 4a), but this difference was statistically not significant (*p* = 0.07, one-way ANOVA followed by Tukey’s multiple comparison test). In addition, we observed an early and enhanced upregulation of IFN-β gene expression for both materials compared to STV PR8 infection alone at 6 hpi (*p* < 0.0001, two-way ANOVA followed by Dunnett´s multiple comparison test) (Fig. 4b). This early stimulation may explain part of the inhibitory effect during OP7 chimera DIP co-infection in Calu-3 cells. (Note: There was not enough purified DIP material available that was produced at 32 °C and MD at 48 hpi to perform an analysis.)

In summary, the transfer of production from a SF to a STR resulted in similar HA titers, purity and very comparable interfering efficacies of OP7 chimera DIP harvests. SXC purification of the material obtained from STR resulted in a higher in vitro interfering efficacy in MDCK(adh) but not in Calu-3 cells. These results indicate that further scale-up to higher reactor volumes (e.g., industrial scale) should be easily accomplished.

### Perfusion mode production in a bioreactor leads to high cell concentrations, superior yields, and high OP7 chimera DIP purity

Next, we evaluated the possibility of process intensification by cultivation in perfusion mode for OP7 chimera DIP production to achieve higher cell concentrations and thus, higher total virus yields (Bissinger et al. 2019; Wu et al. 2021). Therefore, we implemented a perfusion system using an ATF2 system (Hein et al. 2021b).

**Fig. 5:**
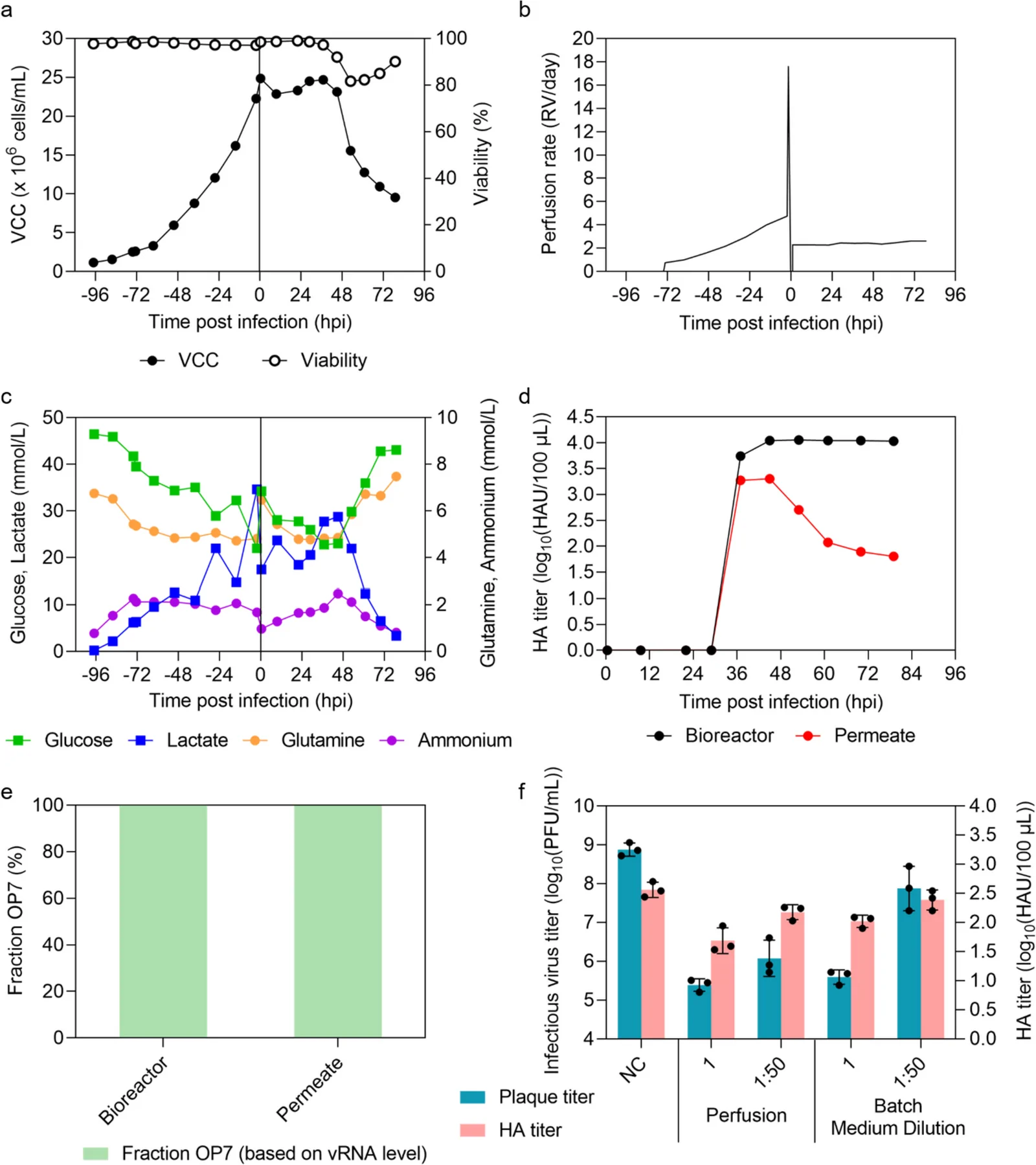
Production of OP7 chimera DIPs in a 1 L STR (700 mL V_W_) in perfusion mode. Inoculation of MDCK-PB2(sus) cells at 1.2 × 10^6^ cells/mL, perfusion start after 24 h of batch mode by using an alternating tangential flow filtration system (ATF2) with a hollow fiber membrane (0.2 μm pore size). Perfusion rate during cell growth controlled by capacitance probe measuring of VCC. Before infection at a MOI of 10^–4^, one RV was exchanged with fresh medium and temperature was reduced to 32 °C. **a)** VCC. **b)** Perfusion rate. **c)** Glucose, lactate, glutamine and ammonium concentration. **d)** HA titer. **e)** Fraction of OP7 chimera DIPs. **a-e** depict the results of one experiment. The optimal harvest time point (45 hpi) was analyzed for **e** and **f**. **f)** Interference assay with MDCK(adh) cells. For comparison, material produced in batch mode using medium dilution (MD) (STR 1) is shown (Fig. 3). Interference assay was performed in three independent experiments. Error bars indicate the SD

Cells were seeded at 1.2 × 10^6^ cells/mL into the STR (700 mL V_W_) (Fig. 5a) and perfusion mode was initiated 24 h after inoculation. During the cell growth phase (-97 to -2 hpi), a cell-specific growth rate of 0.031 h^−1^ was achieved, which is comparable to batch production with MD in STR (0.032–0.036 h^−1^) (Fig. 3a). In addition, viability remained above 97% (Fig. 5a). This indicates that the use of an ATF2 system has no negative impact on cell growth and survival. During the cell growth phase, the perfusion rate was controlled at a predefined CSPR of 200 pL/cell/day. The linear regression of the offline measured VCC and the online permittivity signal during the cell growth phase showed a *R*^2^ of 0.997 (Fig. S4).

After 97 h, cells were infected at 24.9 × 10^6^ cells/mL (as suggested by Hein et al. (Hein et al. 2021b) at a MOI of 10^–4^. Before infection, one RV was exchanged with fresh medium (Fig. 5b and c) by employing an average perfusion rate of 17.6 RV/day for 1 h; in addition, the temperature was lowered to 32 °C. Following infection, the perfusion rate was set at 0 RV/day for 1 h to avoid virus particle wash-out. Subsequently, medium was fed constantly (2.4 RV/day, increased to 2.6 RV/day at 30 hpi) (Fig. 5b). Over process time, neither a glucose nor a glutamine limitation was detected (Fig. 5c). Maximum lactate and ammonium concentrations were 34.7 mmol/L and 2.5 mmol/L, respectively (Fig. 5c). After infection, VCC remained constant until 37 hpi, after which cell lysis started (Fig. 5a). At 45 hpi, the HA titer peaked with 4.04 log_10_(HAU/100 µL) in the bioreactor vessel (Fig. 5d). Also note that until time of optimal harvest part of the virus particles passed the pores of the hollow fiber membrane (0.2 μm) (P_Perm_ = 26%) (Fig. 5d) resulting in an accumulated HA titer (HA_acc_) of 4.10 log_10_(HAU/100 µL). This corresponded to more than 14-fold higher total virus yields compared to the STRs operated in batch mode with MD (all below 3 log_10_(HAU/100 µL)) (Fig. 3b). After time of optimal harvest, decreasing total virus titers were observed in the permeate line (Fig. 5d), likely due to membrane fouling. Importantly, no cells passed the hollow fiber membrane (data not shown).

Table 1 summarizes HA_acc_, the total number of produced virus particles (vir_tot_), CSVY, space–time yield (STY) and volumetric virus productivity (VVP), which were all increased compared to the STR batch process performed at 32 °C and MD, except for the VVP. Further, these coefficients were slightly increased for the perfusion process when virus particles in the permeate line were taken into account as well, relative to harvesting the bioreactor vessel alone. In addition, very high OP7 chimera DIP fractions (99.8%) were present in both the bioreactor vessel and permeate line (Fig. 5e). Ultimately, the in vitro interfering efficacy was evaluated in MDCK(adh) cells (Fig. 5f). At a dilution of 1:50, the material produced in the perfusion culture showed a significantly higher reduction of the infectious virus particle release compared to the batch process with MD (STR 1, Fig. 3) (*p* < 0.001, one-way ANOVA followed by Tukey’s multiple comparison test).

**Table 1:** Summary of OP7 chimera DIP production in a 1 L STR in batch mode (with medium dilution) and perfusion mode

	Spent medium^a^	VCC_max_^b^	HA_acc_^c^	vir_tot_^d^	CSVY^e^	STY^f^	VVP^g^
	mL	× 10^6^ cells/mL	log_10_(HAU/100 µL)	virions	virions/cell	log_10_(virions/L/day)	log_10_(virions/L/day)
Batch STR 1	692	3.3	2.94	1.2 × 10^13^	5289	12.6	12.6
Batch STR 2	691	3.0	2.91	1.1 × 10^13^	5423	12.6	12.6
Batch STR 3	707	2.8	2.74	7.9 × 10^12^	4039	12.3	12.3
Perfusion STR4 B^h^	8804	24.9	4.04	1.5 × 10^14^	8789	13.6	12.5
Perfusion STR4 B + P^i^	8804	24.9	4.10	1.9 × 10^14^	10,648	13.7	12.6

^a^Batch: V_W_ at TOI. Perfusion: V_W_ plus spent medium volume until time of optimal harvest (45 hpi)

^b^Maximum VCC (VCC_max_) after TOI

^c^Accumulated HA titer (HA_acc_) according to Eq. 1

^d^Total number of produced virus particles (vir_tot_) was derived from the HA titer according to Eq. 3

^e^Cell-specific virus yield (CSVY) was derived from the HA titer according to Eq. 4

^f^Space-time yield (STY) was derived from the HA titer according to Eq. 5

^g^Volumetric virus productivity (VVP) was derived from the HA titer according to Eq. 6

^h^Calculations based only on virus particles in the bioreactor vessel

^i^Calculations based on virus particles in the bioreactor vessel plus in the permeate

Overall, we demonstrate the successful establishment of a perfusion process for cell culture-based production of OP7 chimera DIPs free of contaminating infectious STV. Besides, an increase in total virus yields and a CSVY exceeding those of conventional batch processes and very high purity of OP7 chimera DIPs (99.8%) was obtained.

## Discussion

IAV DIPs are regarded as a highly interesting option for future broad-spectrum antiviral therapy (Dimmock et al. 2008, 2012b; Easton et al. 2011; Huo et al. 2020; Kupke et al. 2019; Rand et al. 2021; Scott et al. 2011; Zhao et al. 2018). We recently established a cell-culture-based production of OP7 chimera DIPs together with cDIPs in the absence of infectious STVs. Yet, only relatively low total virus yields and OP7 chimera DIP fractions were achieved for production in shake flasks (Dogra et al. 2023). Here, we present results for scalable processes in laboratory-scale STRs including batch- and perfusion mode strategies that yielded up to a 79-fold increase (perfusion) in total virus yields compared to an original batch process in shake flasks. In addition, we demonstrate the production of almost pure OP7 chimera DIP preparations (up to 99.8%), which is advantageous with respect to regulatory requirements for GMP production towards clinical development.

### Effect of temperature reduction on DIP titers, purity and interfering efficacy

Our data confirms other studies reporting that a temperature reduction during the virus production phase can increase IAV yields (Fig. 2b) (Hein et al. 2021b; Wu et al. 2021). Similar findings were obtained for vesicular stomatitis virus (VSV) (Elahi et al. 2019), Newcastle disease virus (NDV) (Jug et al. 2023) and recombinant adenovirus (Jardon and Garnier 2003). In contrast, other studies did not see a positive effect of temperature reduction on the replication of viruses, e.g., for recombinant VSV-NDV (Göbel et al. 2023) and yellow fever virus (YFV) production (Nikolay et al. 2018). Furthermore, a reduction of temperature during virus production might also be beneficial regarding virus degradation as shown for YFV, Zika virus and IAV (Nikolay et al. 2018; Petiot et al. 2011). At lower temperatures, enzyme activities are reduced and the degradation of infectious virus particles by, e.g., proteases released by lysed cells, can be partly prevented. Eventually, a reduction in temperature to 32°C can support a shift in cellular metabolism, resulting in a reduced accumulation of ammonium, lactate and other inhibitory metabolites released in the supernatant. In our study, increased concentrations of ammonium (> 4 mM) were likely associated with lower total virus yields for OP7 chimera DIP production at 37 °C (Fig. S1d). This is in line with a review reporting that ammonium and lactate concentrations at 2–3 mM and above 20–30 mM, respectively, can affect cell growth and virus yield, depending on the cell line (Schneider et al. 1996). The higher purity of OP7 chimera DIPs (up to 99.8%) for all performed runs at 32 °C might be explained by increased virus replication. As a result, OP7 chimera DIPs likely overgrew Seg 1 cDIPs due to the replication advantage of Seg 7-OP7 vRNA. The higher interfering efficacy of material produced with MD at 32 °C relative to 37 °C can be attributed to the higher total virus yield and fraction of OP7 chimera DIPs.

Previously, we showed for cultivations with CME performed at 37 °C that production and interfering efficacy of OP7 chimera DIPs was highly dependent on MOI (Dogra et al. 2023). For MD and 32 °C, however, total virus titers, OP7 chimera DIP fraction and interfering efficacy were almost not affected by MOI. This suggests that the selection of the optimal MOI is less important under this production condition, and process robustness could be improved. Using lower MOIs for production could reduce costs required for seed virus generation.

### Process intensification using perfusion mode cultivation

Through process intensification, we achieved a high total virus yield of the OP7 chimera DIPs in perfusion culture (24.9 × 10^6^ cells/mL) with strongly increased total number of virus particles and STY (up to 23-fold) relative to the batch process (2.8–3.3 × 10^6^ cells/mL), while VVP was comparable (Table 1). Moreover, we produced similar yields of virus particles (4.10 log_10_(HAU/100 µL), a CSVY of 10648 virions/cell, 24.9 × 10^6^ cells/mL, 32 °C) compared to a production of STV IAV in perfusion mode with a different suspension MDCK cell line derived from an adherent MDCK cell line originating from the American-Type Culture Collection (ATCC, MDCK ATCC CCL-34) (≥ 4.37 log_10_(HAU/100 µL), ≥ 9299 virions/cell, ≥ 43 × 10^6^ cells/mL, 33 °C) (Wu et al. 2021). An often described phenomenon in virus production is the so-called “cell density effect”—a reduction of CSVY with an increase in VCC (Nadeau and Kamen 2003). This effect is often attributed to the exhaustion of nutrients and the accumulation of inhibitory by-products of metabolism including ammonium or lactate, but can be prevented by cultivation in perfusion mode (Bock et al. 2011; Genzel et al. 2014; Henry et al. 2004). The about 2-fold higher CSVY compared to the batch process (Table 1) confirmed that the “cell density effect” is not relevant for perfusion mode cultivations. Clearly, the relatively high CSPR (200 pL/cell/day) and the exchange of one RV with fresh medium prior to infection was sufficient to prevent the depletion of substrates and avoid the accumulation of ammonium and lactate as inhibiting metabolic by-products. Similar results were already reported for the production of DI244, a well-known cDIP of IAV (Dimmock et al. 2008, 2012a; Hein et al. 2021a) using the same cell line (Hein et al. 2021b). Furthermore, for other suspension MDCK cells (ATCC) cultivated in the same medium in perfusion culture, a CSPR of only 40–60 pL/cell/day was sufficient to achieve good process performance (Wu et al. 2021). Although, the applied high perfusion rate resulted in higher costs, the increased STY achieved relative to the batch process possibly should help to overcome this disadvantage (Göbel et al. 2022). Nevertheless, additional studies should be performed regarding optimal setting of the CSPR for manufacturing at final process scale.

During the cell growth phase, the perfusion rate was controlled using a capacitance probe to improve process robustness and reduce medium use as already demonstrated for other cell lines (Gränicher et al. 2021; Hein et al. 2021b; Nikolay et al. 2018; Wu et al. 2021). Recent studies reported that the presence of trypsin in the virus production phase influences the permittivity signal (Petiot et al. 2017; Wu et al. 2021). To avoid an interference of trypsin on the perfusion rate control, which is based on the permittivity signal, we decided to set a constant perfusion rate after virus infection as also done by others (Hein et al. 2021b; Vázquez-Ramírez et al. 2018).

Filter fouling is a typical phenomenon to be considered for use of retention devices including hollow fiber membranes (Genzel et al. 2014; Hein et al. 2021b; Nikolay et al. 2020). For virus retention, not only the nominal pore size, but also the membrane material itself plays a crucial role (Nikolay et al. 2020). Furthermore, the temperature during production can affect virus retention. For IAV production at 37 °C, only a very low fraction of virus particles passed a PES hollow fiber membrane (0.2 μm pore size) (Wu et al. 2021) as expected (Genzel et al. 2014). However, reducing the temperature to 33°C at TOI allowed harvesting of a considerable percentage of virus particles via the permeate (Wu et al. 2021), as also shown in our study (P_Perm_ = 26%) at a production temperature of 32 °C. In contrast, for the production of DI244, virus particles did not seem to pass the hollow fiber membrane at 32 °C. However, virus quantification in the referred study was only carried out at very late time points of production in the permeate line, so that number of virus particles passing the membrane most likely was underestimated largely (Hein et al. 2021b). Nevertheless, filter fouling could not be prevented at later time points in our study (Fig. 5d). Recently, a novel tubular membrane (about 10 μm pore size, Artemis Biosystems) with an ATF-2 system was successfully tested for continuous virus harvesting of DI244 with a very high cell retention efficiency (Hein et al. 2021b). Continuous virus harvesting was also demonstrated by using the Tangential Flow Depth Filtration system (TFDF, Repligen) for lentiviral vector (Tona et al. 2023; Tran and Kamen 2022) and adeno-associated virus (Mendes et al. 2022) production in perfusion mode. In general, continuous virus harvesting through a membrane that allows for direct cooling of produced virus material with a first clarification improves virus stability and, therefore, yields. The use of an acoustic settler (Gränicher et al. 2020; Henry et al. 2004) or an inclined settler (Coronel et al. 2020) would be alternative options. Regarding the former, a more than 1.5-fold higher CSVY and VVP compared to an ATF system with a PES hollow fiber membrane (0.2 μm pore size) was be obtained for harvesting IAV (Gränicher et al. 2020). For the production of OP7 chimera DIPs in perfusion mode, the use of a membrane or the implementation of another perfusion system that allows for continuous virus harvest over the complete production time would likely be beneficial and should be envisaged in the design and optimization of a GMP-ready manufacturing process.

Overall, a scalable and high-yield cell culture-based production process in perfusion mode for OP7 chimera DIPs not contaminated with infectious STV and almost free of Seg 1 cDIPs is now available. Together with the encouraging data obtained from recent animal studies of OP7 chimera DIPs, this paves the way towards GMP-process development and clinical studies.

## Supplementary Information

Below is the link to the electronic supplementary material.

Supplementary file1 (PDF 441 KB)

## Data Availability

The datasets generated during and/or analyzed during the current study are available from the corresponding author upon request.
