# Supplementary material for: Production of antiviral “OP7 chimera” defective interfering particles free of infectious virus
Source: Appl Microbiol Biotechnol. 2024 Jan 13;108:97. doi: 10.1007/s00253-023-12959-6 (PMC10787692; doi:10.1007/s00253-023-12959-6)
Supplement: Supplementary file 1 [file 253_2023_12959_MOESM1_ESM.pdf]

Journal: "Applied Microbiology and Biotechnology"

Manuscript title: "Production of antiviral "OP7 chimera" defective interfering particles free of infectious virus"

Lars Pelz[1], Tanya Dogra[1], Pavel Marichal-Gallardo[1], Marc Dominique Hein[2], Ghada Hemissi[1], Sascha Young Kupke[1,*], Yvonne Genzel[1,*], Udo Reichl[1,2]

[1]Max Planck Institute for Dynamics of Complex Technical Systems, Bioprocess Engineering, Magdeburg, Germany

[2]Otto von Guericke University Magdeburg, Bioprocess Engineering, Magdeburg, Germany

***Correspondence:**

Sascha Y. Kupke

kupke@mpi-magdeburg.mpg.de

Yvonne Genzel

genzel@mpi-magdeburg.mpg.de

Supplementary Material

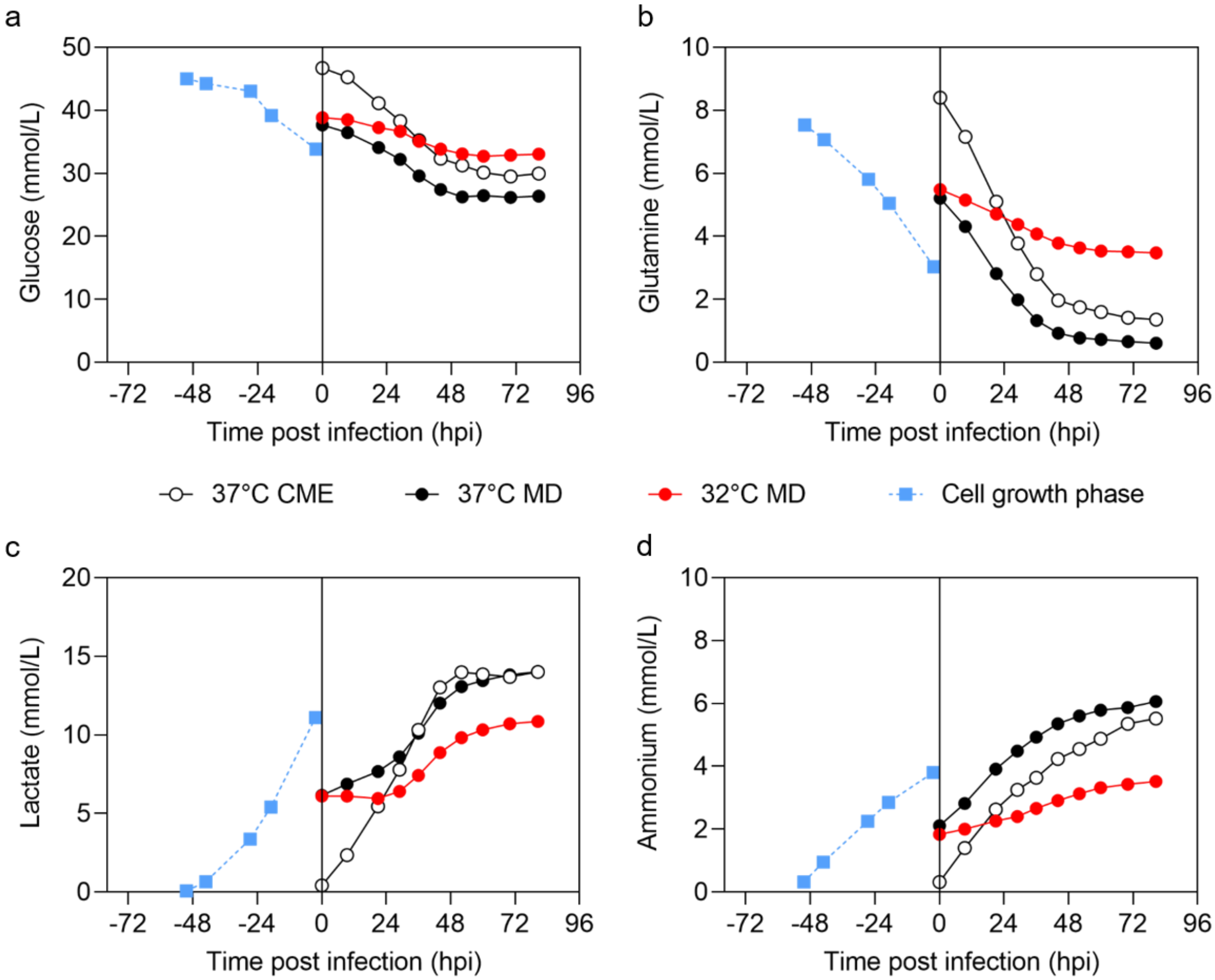

Fig. S1 Extracellular metabolite concentrations for batch-mode production of OP7 chimera DIPs in shake flasks (Fig. 2). **a)** Glucose, **b)** glutamine, **c)** lactate, and **d)** ammonium concentrations. MDCK-PB2(sus) cells, cultivated in 250 mL shake flasks (100 mL V_W) at 37°C, were grown to about 4.0×10^6 cells/mL. Subsequently, the suspension culture was diluted (MD, 1:2) with fresh medium (100 mL V_W), cells were infected at a MOI of 1E 4 and temperature was reduced to 32°C. For comparison, two cultivations were performed at 37°C, one with MD at an optimal MOI of 1E-3, one with complete medium exchange (CME) at an optimal MOI of MOI 1E-4.

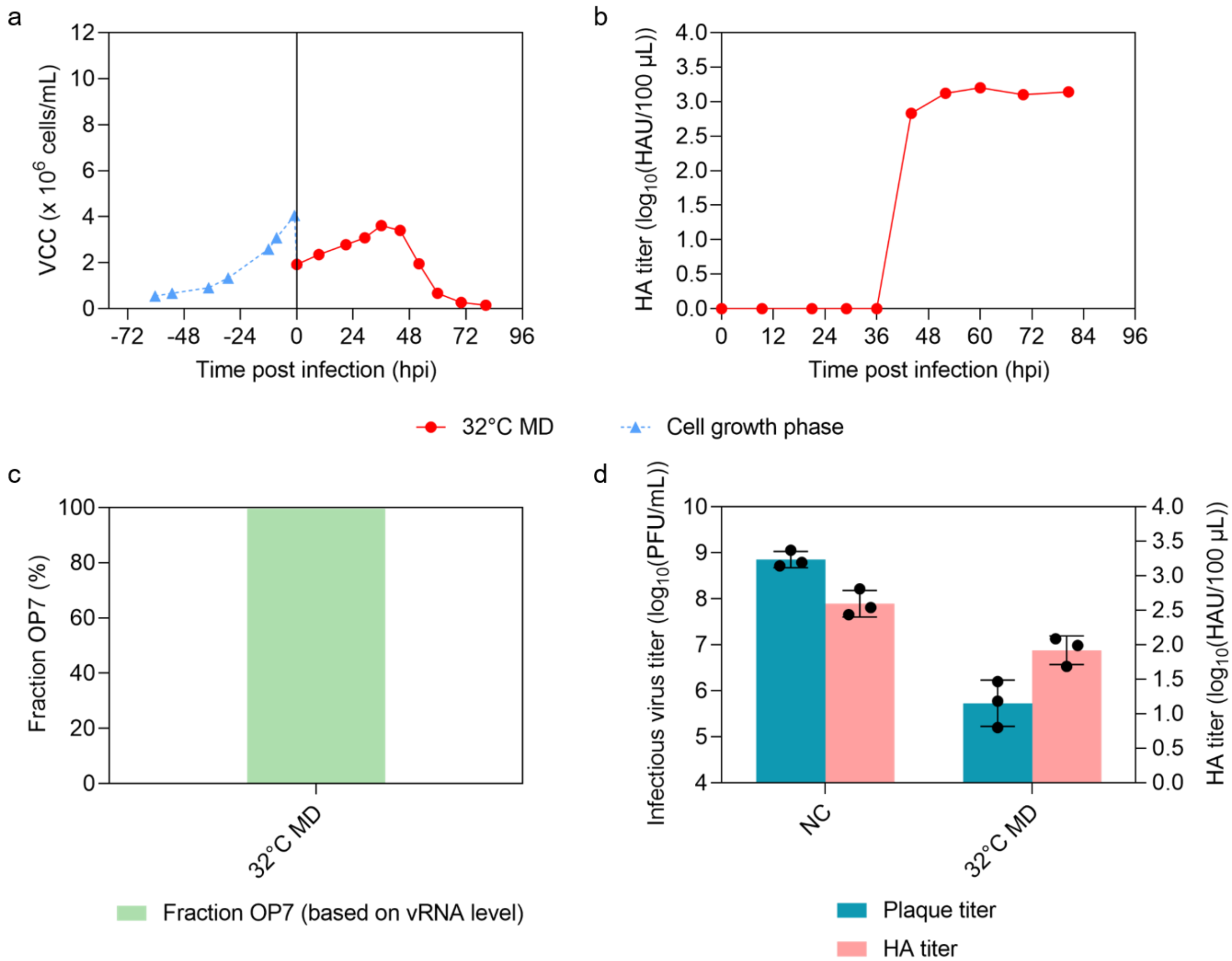

Fig. S2 Second independent batch-mode production of OP7 chimera DIPs in shake flasks**.** MDCK-PB2(sus) cells, cultivated in 250 mL shake flasks (100 mL V_W) at 37°C, were grown to about 4.0 × 10^6 cells/mL. Subsequently, the suspension culture was diluted (MD, 1:2) with fresh medium (100 mL V_W), cells were infected at a MOI of 1E 4 and temperature was reduced to 32°C. **a)** VCC. **b)** HA titer. **c)** OP7 chimera DIP fraction. **a, b** and **c** depict the results of one experiment. The optimal harvest time point was analyzed for **c** and **d** (32°C MD: 44 hpi). **d**) Interference assay with MDCK(adh) cells was performed in three independent experiments. Error bars indicate the SD.

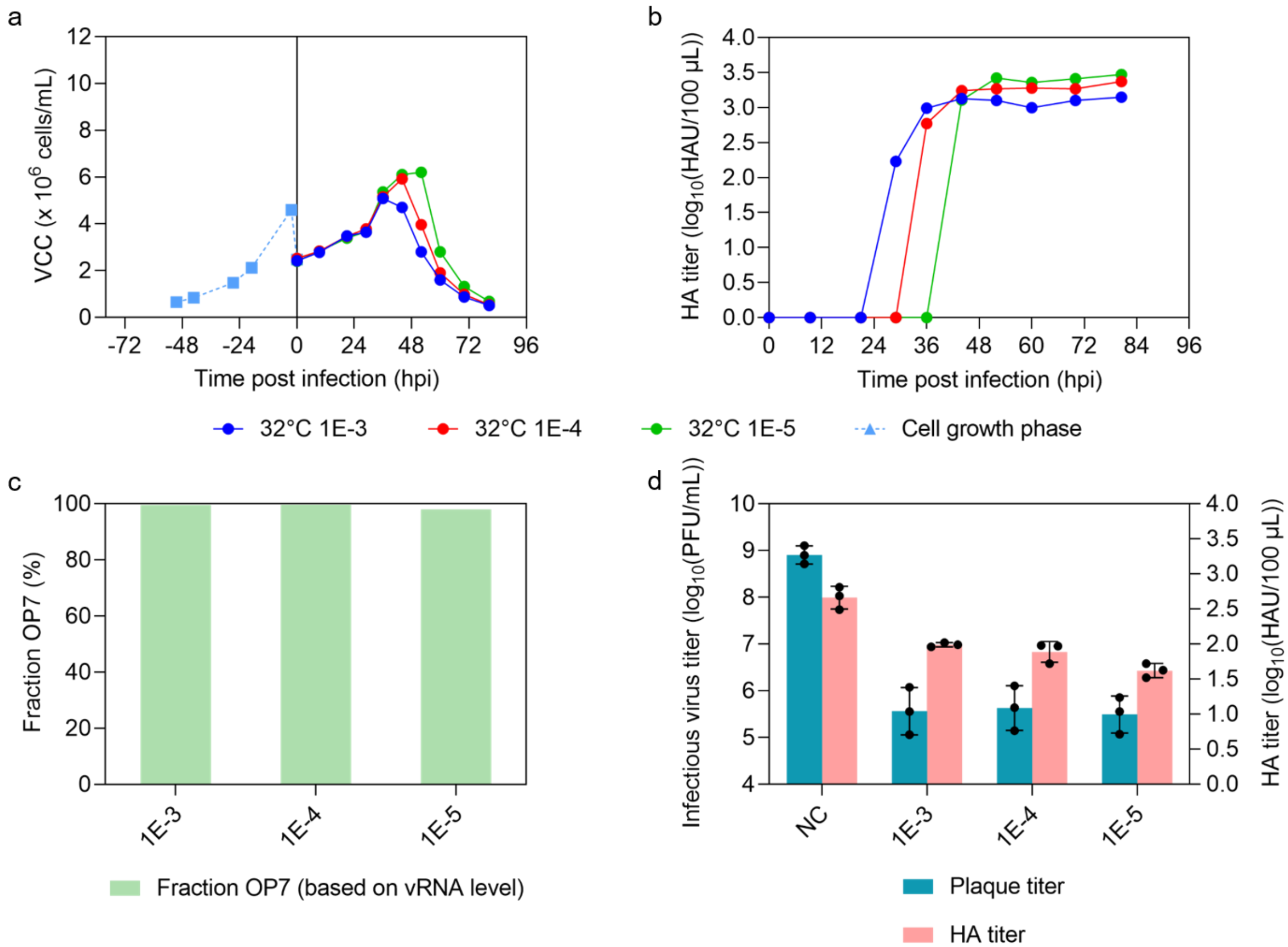

Fig. S3 Batch-mode production of OP7 chimera DIPs in shake flasks (also see Fig. 2) at three different MOIs. MDCK-PB2(sus) cells, cultivated in 250 mL shake flasks (100 mL V_W) at 37°C, were grown to about 4.0×10^6 cells/mL. Subsequently, the suspension culture was diluted (MD, 1:2) with fresh medium (100 mL V_W), cells were infected at a MOI of 1E-3, 1E-4 or 1E-5 and temperature was reduced to 32°C. **a)** VCC. **b)** HA titer. **c)** Fraction of OP7 chimera DIPs. **a-c** depict the results of one experiment. **d)** Interference assay with MDCK(adh) cells was performed in three independent experiments. Error bars indicate the SD. The optimal harvest time point (1E-3: 36 hpi, 1E-4: 44 hpi; 1E-5: 52 hpi) was analyzed for **c** and **d**.

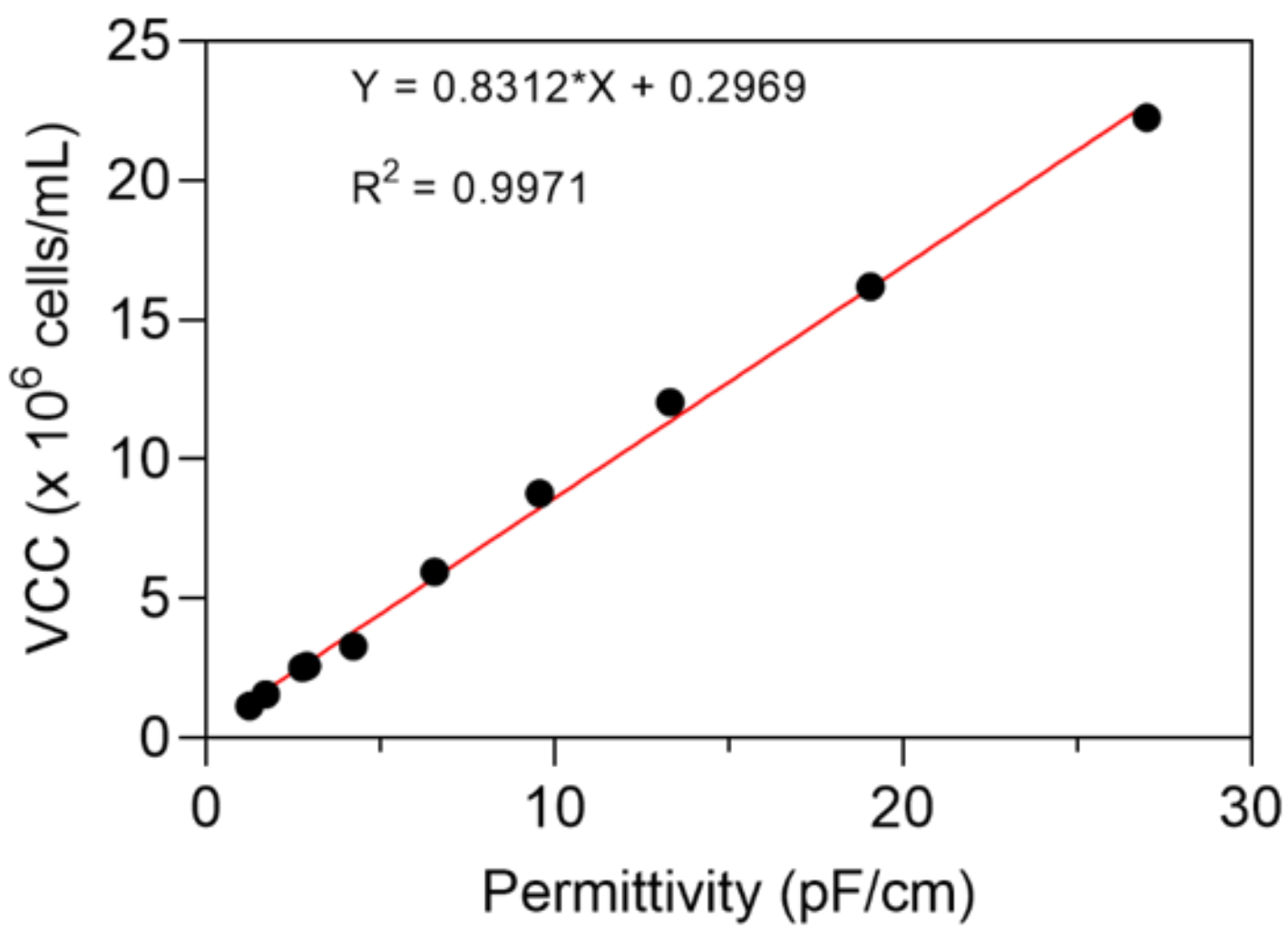

Fig. S4 Linear regression of VCC and permittivity signal during OP7 chimera DIP production in perfusion culture. MDCK-PB2(sus) cells were grown in a 1 L stirred tank bioreactor (700 mL V_W). Perfusion started after 24 h of batch-mode using an alternating tangential flow filtration system (ATF2) with a hollow fiber membrane. The estimated VCC was used to the control perfusion rate during cell growth.
